# Veno-venous extracorporeal membrane oxygenation successfully treated a case of severe pulmonary hemorrhage caused by leptospirosis

**DOI:** 10.1186/s12879-020-05518-1

**Published:** 2020-10-27

**Authors:** H. J. Wang, G. Z. Chen, C. J. Zhou, Y. FU, L. N. YAO

**Affiliations:** Department of Intensive Care Unit, the Affiliated People’s Hospital of Ningbo University, 251 East Baizhang Road, Ningbo City, 315010 Zhejiang Province P.R. China

**Keywords:** Leptospirosis, Pulmonary hemorrhage, ARDS, ECMO, Case report

## Abstract

**Background:**

Pulmonary hemorrhage is an important complication of leptospirosis. Once acute respiratory distress syndrome (ARDS) occurs as a secondary condition, treatment is extremely difficult and the mortality rate is very high.

**Case presentation:**

The patient was a 49-year-old. He was admitted to the hospital because he had experienced a fever and cough for 4 days. Hemorrhage, respiratory failure, ARDS and other symptoms appeared soon after admission. Due to severe pulmonary hemorrhage secondary to ARDS, mechanical ventilation was performed through tracheal intubation. During intubation, the patient suffered cardiac arrest, and the patient’s condition worsened. He was confirmed to have leptospirosis through second-generation sequencing of the alveolar lavage fluid. Finally, we successfully treated the patient with penicillin as an anti-infective medication and venous-venous extracorporeal membrane oxygenation (v-vECMO). To the best of our knowledge, this report is the first to describe the successful application of ECMO in mainland China.

**Conclusions:**

Leptospirosis can induce serious but transient ARDS with a better prognosis than other causes of ARDS. Our patient was successfully treated with V-vECMO.

## Background

Leptospirosis is a zoonotic infectious disease caused by Leptospira. Leptospiral infection has a broad spectrum of clinical manifestations, ranging from a subclinical or mild illness to a fulminant life-threatening illness. Symptoms suggesting leptospirosis are non-specific but usually include fever, headache and myalgia, sometimes with hemorrhage or meningitis as the initial presentation [1, 2], and the disease has an average mortality rate of 6.85%. However, once complicated by pulmonary hemorrhage secondary to acute respiratory distress syndrome (ARDS), the mortality rate increases to 51 to 100% due to the lack of effective treatment [3, 4]. Use ECMO as a potential treatment modality with leptospirosis is increasing. However, this method requires advanced technology and is expensive. Currently, case reports are scarce and have not been widely conducted, particularly in developing countries. To our knowledge, no similar studies have been conducted in mainland China, particularly in patients with secondary cardiac arrest. The report of the successful treatment of secondary cardiac arrest induced by severe intravenous hemorrhagic leptospirosis with v-vECMO in our hospital is described below.

## Case presentation

**Fig. 1:**
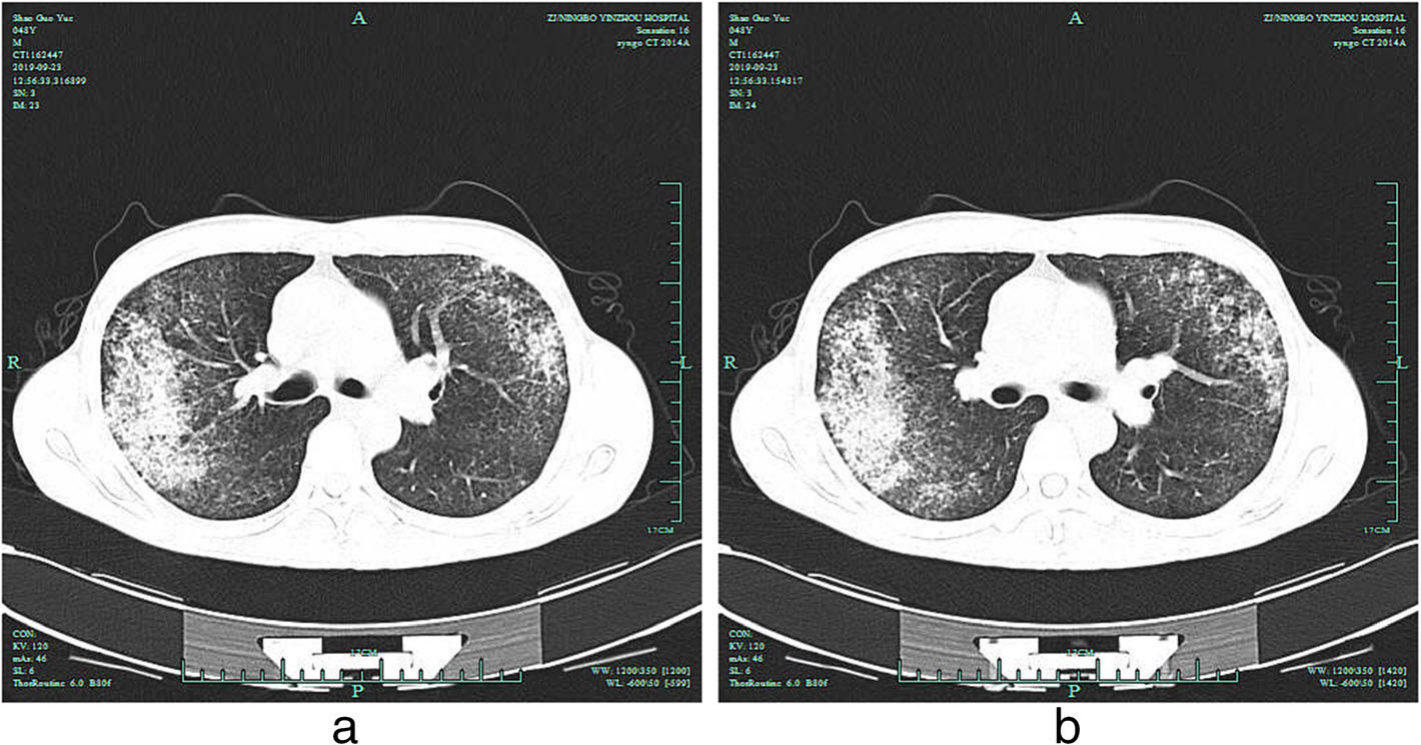
Chest CT on the day of admission. Both lungs presented exudative lesions, which are obvious in the image of the right lung

**Fig. 2:**
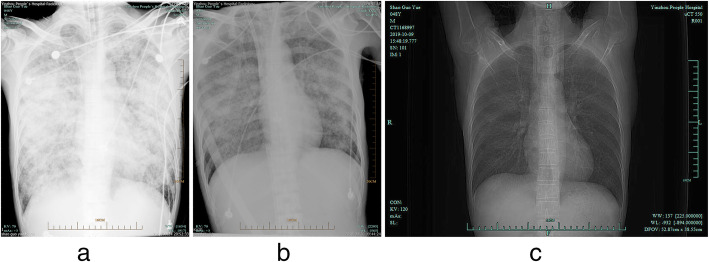
**a**: Chest X-ray before ECMO treatment. Diffuse exudation was observed in both lungs, presenting as a large white lung. **b**: Chest X-ray on day 6 of ECMO treatment. The diffuse exudation in both lungs was significantly absorbed compared with the image captured at admission. **c**: Chest X-ray on the 9th day after the removal of ECMO. The pneumonia was completely absorbed without residual disease

The patient was a 49-year-old self-employed male in good health who liked to tend to flowers and plants and was found to show evidence of mouse bites. The patient developed a cough at home 4 days prior to admission. Occasionally, the sputum was tinged with red blood, which was accompanied by pharyngeal and knee pain; his highest temperature was 39.2 °C and was accompanied by chills. He did not experience night sweats, herpes of the mouth, chest pain, abdominal pain, diarrhea, hoarseness, headache, vomiting, etc. He visited our hospital because of chest tightness after activity. Upon admission, the patient was conscious, with a temperature of 37.5 °C, a pulse rate of 105 beats/min, a respiration rate of 18 breaths/min, and a blood pressure of 100/49 mmHg. The following observations were recorded: the bulbous conjunctiva was free of congestion, his lips were not bluish, wet rales were heard in both lungs, his heart rate was 105 beats/minute. The laboratory tests showed a c-reactive protein level of 90.87 mg/L, and white blood cell count, 9.6 × 10^9^ /L; hemoglobin, 117 g/L,platelet count, 96 × 10^9^ /L. The results of the blood gas analysis revealed a PO_2_ of 119 mmHg, pH of 7.43; PCO_2_ of 38 mmHg, and procalcitonin (PCT) level of 4 pg/mL (Table 1). The chest CT revealed bilateral pulmonary infiltrations (Fig. 1). After admission, piperacillin tazobactam was administered at a dose of 4.5 g iv q8h due to moderate severe pneumonia, a 40 mg methylprednisolone injection was administered to control body temperature, and supportive nasal catheter oxygenation was provided (5 L/min). The patient’s condition worsened after 4 h, with a temperature increased to 39 °C, a heart rate of 150 beats/min, a respiratory rate of 26 breaths/min, a blood pressure of 85/55 mmHg, shortness of breath, but no chills; additionally, the patient’s SpO_2_ level through the nasal catheter decreased to 70%. The patient’s SpO_2_ increased to 86%, when switched to a venturi mask, but his shortness of breath persisted. The patient was transferred to ICU for adequate fluid resuscitation and blood culture is collected. The patient soon exhibited hemoptysis, his SpO_2_ decreased to 67%, the PO_2_ of 43 mmHg, blood pressure was 96/56 mmHg, and wet rales in both lungs had significantly increased. A pulse index continuous cardiac output examination was performed, which resulted in a extravascular lung water index of 36.9 mL/kg (normal range 3.0–7.0) and pulmonary vascular permeability index of 6.0 dn.s.m.^2^/cm^5^ (normal range 1.0–3.0). The patient’s heart function was normal, his lung permeability increased, and the presence of extravascular fluid in the lung increased. After 10 h in the ICU, the patient’s condition deteriorated again. He was agitated under noninvasive mechanical ventilation, and the SpO_2_ level was difficult to maintain within the normal range. Mechanical ventilation via tracheal intubation was performed. During the process of endotracheal intubation, a large amount of bloody secretions sprouted from the airway, the heart rate rapidly decreased to 40 beats/min, and the arterial pulse and SpO_2_ were not detected; Cardiopulmonary resuscitation was implemented immediately. After 1 min of cardiopulmonary resuscitation, the patient recovered spontaneous circulation but was still agitated and in respiratory distress with an SpO_2_ of 80%, despite full mechanical ventilator support. Five hours after the patient was sedated using midazolam and fentanyl through continuous micropumping, his shortness of breath was still not relieved. The ventilator provided a small tidal volume ventilation with a frequency of 50–60 breaths/min. The blood gas analysis indicated a pH of 7.19, pCO_2_ of 65 mmHg, PO_2_ of 61 mmHg, and hemoglobin concentration of 68 g/dL. The patient showed significant pulmonary hemorrhage and clinical manifestations of ARDS. After a discussion among the staff of the cardiology department, thoracic surgery department, ICU, respiratory department and other experts in our hospital, v-vECMO was initiated at 4.5 L/min, with sweeping gas flow through the oxygenator at 4.5 L/ min of 100% oxygen. After the procedure, the patient’s SpO_2_ was 100%. The partial thromboplastin time (PTT) was maintained at 40–50 s by administering a heparin infusion, and the PTT was monitored every 4 h to avoid aggravating the pulmonary hemorrhage. The ECMO blood flow was maintained at 4.5 L/min during the first 3 days. Because general community-acquired pneumonia was considered an unlikely diagnosis, 4.5 g of piperacillin tazobactam iv q8h and 0.5 g of azithromycin iv qd as anti-infection medications and 40 mg of methylprednisolone q8h were continued. A tracheoscopic examination prompted diffuse bleeding in the airway and alveolar lavage fluid was collected and for second-generation gene sequencing on the next day. The hemoglobin concentration decreased to 61 g/dL after an infusion with 3 units of red blood cells, and the platelet count decreased to 57 × 10^9^/L. Six units of a red blood cell suspension were applied to correct the anemia, and 10 units of platelets and 600 mL of fresh frozen plasma were re-administered to supplement the clotting factors. The laboratory tests revealed an increase in the PCT level to 77 ng/mL, and the antibiotics were upgraded to 0.5 g of intravenous meropenem q6h for a suspected severe infection. Four days later, second-generation gene sequencing of the patient’s alveolar lavage fluid was positive for *Leptospira interrogans*. Although the blood culture and urine culture did not detect leptospirosis, prior to discharge, the results showing strongly positive *Leptospira* serology with an IgM titer of 1:1280 detected using an enzyme-linked immunosorbent assay (ELISA). Leptospirosis was considered, and the patient was administered an injection of 800,000 U of penicillin q8h (with a first dose of 400,000 U) as anti-infective treatment. The patient was sedated using midazolam and fentanyl during the ECMO supportive treatment, and no obvious complications occurred. After 6 days, the chest radiograph of the patient was significantly improved and ECMO was stopped (Fig. 2). On day 9, the endotracheal tube was removed, and the patient was transferred to the general ward on day 10.

**Table 1:** Laboratory examination results during ICU hospitalization

Days	Normal range	0	1	2	3	4	5	6	7	8	9	10	11
WBCx109/L	3.5–9.5	6.4	7.8	10.3	9.7	9.9	11.7	14.2	20.1	23.3	25.3	30.7	20
hemoglobin (g/L)	115–150	117	64	90	102	81	93	99	121	118	117	128	127
platelet (× 109/L)	125–350	96	57	102	118	170	170	197	164	174	186	231	250
PCT (ng/mL)	0.02–0.05	4.7	74	29	12.8	12.8	3.28	1.96	0.95	0.62	0.57	0.4	0.22
CRP (mg/dL)	0–8.0	93	119.6	119	23	16	14	6.69	4.07	6.31	3.51	26.14	53.2
CREA (umol/L)	41–81	90	108	79	87	62	54	46	42	46	42	39	48
AST(u/L)	13–35	26	24	25	28	27	43	51	62	38	28	16	14
ALT(u/L)	7–40	98	93	115	132	150	169	173	135	73	52	37	38
TBIL (umol/L)	3.1–17.1	14.1	41.3	34.7	29	20.3	27.7	24.2	21.2	19.6	17.6	12.3	14.3
PT (s)	9.2–13.1	14.4	29.4	18.4	19.3	23.5	31.3	140	16.6	14.8	12.7	16.5	13.1
APTT (s)	25.4–40.9	27.5	28.9	61.5	40.4	46.4	38.4	38.4	33.3	32.6	32.1	27.5	26.9
INR	0.82–1.15	1.1	1	1.12	1.11	1.14	1.09	1.14	1.23	1.17	1.1	1.01	1.14
FIB (g/l)	2.0–4.0	1.93	1.94	1.66	1.74	1.96	1.83	2.4	2.92	2,.43	1.93	3.4	3.7

## Discussion and conclusions

Leptospirosis is an animal-transmitted infectious disease that was once widely prevalent in Zhejiang Province. From the mid-1960s to the early 1970s, four great epidemics occurred. After the mid-1970s, the incidence of leptospirosis decreased annually. Since 2000, the annual incidence in the whole province has decreased to less than 0.2/100,000. Some medical personnel have an insufficient understanding of leptospirosis, and the etiology is often difficult to determine, which might easily lead to a missed diagnosis and misdiagnosis. Our medical institution has not diagnosed leptospirosis for more than 20 years, and the relevant laboratory tests have not been routinely performed. The clinical manifestations of leptospirosis vary and include fever, jaundice and acute renal injury. Lung manifestations occur in 20–70% of patients, but are often masked by other symptoms. Severe pulmonary hemorrhage is the main cause of death associated with leptospirosis and usually occurs in 4–6 days; severe pulmonary hemorrhage manifests as extensive interstitial and alveolar hemorrhage, pulmonary congestion, and pulmonary edema. The specific mechanism is unknown. According to Croda et al, the linear deposition of immunoglobulins (IgA, IgG, and IgM) and complement on the alveolar surface may play a role in the pathogenesis [5]. Researchers have posited that a Jarisch–Herxheimer reaction after the first application of antibiotics may be the main cause of disease progression and it particularly aggravates pulmonary hemorrhage [6]. This patient also showed a rapid onset of symptoms 4 h after the application of antibiotics on the fourth day of onset, which was accompanied by severe pulmonary hemorrhage, shock, hyperthermia, etc. The determination of whether the disease itself progresses or if the Jarisch–Herxheimer reaction that occurs after the application of antibiotics causes adverse effects remains a challenge; with the exception of fever, shock is similar to the disease itself, and no specific and objective laboratory evidence is available. This patient had no obvious clinical manifestations of chills. Clinicians have not determined whether the lack of this manifestation is related to the use of methylprednisolone at admission. Some clinicians postulate that hormones prevent the occurrence of the Jarisch–Herxheimer reaction [7]. However, the PCT dramatically increased from a low level within 24 h of the patient’s deterioration and decreased rapidly the next day, which is rare in patients with sepsis. Observations of additional cases are required to determine whether this finding is related to the fact that the Jarisch–Herxheimer reaction is a self-limited response.

Pulmonary hemorrhage is a serious complication of leptospirosis. The accumulation of hemorrhagic fluid in the alveoli can lead to airway obstruction, loss of ventilated blood and acute gas exchange disorders, which may cause patients to experience significant difficulty breathing. Acute respiratory distress includes hypoxemia; despite the timely initiation of conventional mechanical ventilation, hypoxemia may still be difficult to treat and has a high mortality rate [4, 8]. Although some studies have attempted to treat this disease with high doses of hormones, plasma exchange, hemostatic drugs, high-frequency oscillating ventilation and other methods, the effects remain unclear [9, 10]. Despite the report of a successful ECMO application in patients with ARDS [11], few studies have examined whether ECMO is useful for ARDS caused by severe pulmonary hemorrhage in individuals with leptospirosis; conclusive evidence is lacking, particularly while maintaining ECMO, regarding whether the high-intensity anticoagulation required to avoid embolization events will aggravate pulmonary hemorrhage, and this factor is currently the most important concern. Overall, active hemorrhage is still a relative contraindication for ECMO application. In the present study, the patient presented obvious pulmonary hemoptysis and a reduced hemoglobin concentration after admission, and the diagnosis was unclear. The prediction of whether anticoagulation medication during ECMO maintenance would lead to uncontrolled pulmonary hemorrhage was difficult to determine. However, the patient still presented with significant dyspnea symptoms under high-level mechanical ventilation, which was an important reason why the patient required ECMO. Although heparin should be avoided as much as possible during ECMO maintenance in patients with pulmonary hemorrhage [12], we adjusted the anticoagulant intensity to maintain an PTT of 40–50 s and simultaneously injected plasma, platelets, erythrocyte suspensions and other supportive treatment measures. Fortunately, uncontrolled bleeding was not observed during ECMO in this patient. Despite invasive ventilation with a high PEEP, a maximum oxygen supply and the use of muscle relaxants, analgesics and sedatives, the patient was still difficult to treat. V-vECMO was used to temporarily replace the lung function of the patient and adopt protective strategies to avoid ventilator-related lung injury. After 6 days, the treatment was successful, suggesting that as long as the primary disease is treated effectively, heparin is a feasible treatment to maintain the coagulation status in a reasonable range that does not aggravate pulmonary hemorrhage. ECMO allows the patient’s lungs to rest adequately, which effectively facilitates treatment. This effect may be related to the fact that respiratory failure caused by leptospirosis-induced hemorrhaging is simpler and more transient than the condition caused by other diseases [13].

In conclusion, pulmonary haemorrhage is a serious complication of leptospirosis. In recent years, the incidence of leptospirosis has increased. In this article, we confirmed that genetic sequencing of alveolar lavage fluid also effectively diagnoses the disease, thereby avoiding a missed diagnosis due to negative blood culture, urine culture, and sputum culture. This disease has a high mortality rate, and very limited treatment methods are available. With the increasing number of reports of successful treatment using ECMO, this method may be an effective measure to reduce the mortality associated with leptospirosis; however, further prospective large-scale controlled experimental studies are needed to confirm these findings. Finally, this article has some limitations. For example, because this study reports a retrospective analysis and is limited by cost, blood and urine cultures were not further sequenced after blood and urine cultures were negative.

## Data Availability

All data have been presented within the manuscript and in the form of images.

## Abbreviations

ARDS: Acute respiratory distress syndrome
v-vECMO: Venous-venous extracorporeal membrane oxygenation
PCT: Procalcitonin
ICU: Intensive care unit
